# 10,10-Dimethyl­anthrone

**DOI:** 10.1107/S1600536810008524

**Published:** 2010-03-13

**Authors:** Hoong-Kun Fun, Madhukar Hemamalini, B. P. Siddaraju, H. S. Yathirajan, M. S. Siddegowda

**Affiliations:** a X-ray Crystallography Unit, School of Physics, Universiti Sains Malaysia, 11800 USM, Penang, Malaysia; b Department of Chemistry, V. V. Puram College of Science, Bangalore 560 004, India; c Department of Studies in Chemistry, University of Mysore, Manasagangotri, Mysore 570 006, India

## Abstract

In the title compound, C_16_H_14_O, the asymmetric unit consists of three crystallographically independent mol­ecules. The anthracene units are essentially planar, with maximum deviations of 0.165 (1), 0.153 (1) and 0.045 (1) Å in the three mol­ecules. In the crystal structure, mol­ecules are linked *via* inter­molecular C—H⋯O hydrogen bonds. Further stabilization is provided by C—H⋯π inter­actions.

## Related literature

For analytical applications of the title compound, see: Trevelyan (1952 ▶). For related structures, see: Destro *et al.* (1973 ▶); Ghosh *et al.* (1993 ▶); Iball & Low (1974 ▶); Srivastava (1964 ▶); Zhou *et al.* (2004 ▶; 2005 ▶; 2007 ▶). For bond-length data, see: Allen *et al.* (1987 ▶). For the stability of the temperature controller used in the data collection, see: Cosier & Glazer (1986 ▶).
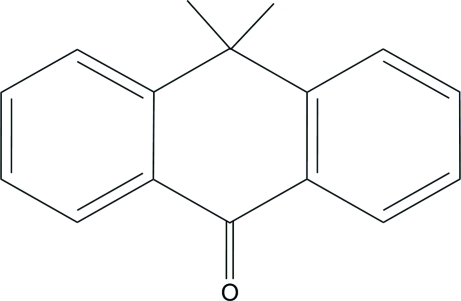


## Experimental

### 

#### Crystal data

C_16_H_14_O
*M* *_r_* = 222.27
Triclinic, 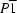

*a* = 11.2438 (6) Å
*b* = 12.1105 (6) Å
*c* = 15.1025 (8) Å
α = 107.955 (1)°
β = 98.734 (1)°
γ = 111.764 (1)°
*V* = 1732.47 (16) Å^3^
*Z* = 6
Mo *K*α radiation
μ = 0.08 mm^−1^
*T* = 100 K
0.62 × 0.31 × 0.27 mm

#### Data collection

Bruker APEX DUO CCD area-detector diffractometer
Absorption correction: multi-scan (*SADABS*; Bruker, 2009 ▶) *T*_min_ = 0.953, *T*_max_ = 0.979
35076 measured reflections
10016 independent reflections
8420 reflections with *I* > 2σ(*I*)
*R*_int_ = 0.028

#### Refinement

*R*[*F* ^2^ > 2σ(*F* ^2^)] = 0.044
*wR*(*F* ^2^) = 0.142
*S* = 1.07
10016 reflections
466 parameters
H-atom parameters constrained
Δρ_max_ = 0.54 e Å^−3^
Δρ_min_ = −0.36 e Å^−3^

### 

Data collection: *APEX2* (Bruker, 2009 ▶); cell refinement: *SAINT* (Bruker, 2009 ▶); data reduction: *SAINT*; program(s) used to solve structure: *SHELXTL* (Sheldrick, 2008 ▶); program(s) used to refine structure: *SHELXTL*; molecular graphics: *SHELXTL*; software used to prepare material for publication: *SHELXTL* and *PLATON* (Spek, 2009 ▶).

## Supplementary Material

Crystal structure: contains datablocks global, I. DOI: 10.1107/S1600536810008524/sj2742sup1.cif

Structure factors: contains datablocks I. DOI: 10.1107/S1600536810008524/sj2742Isup2.hkl

Additional supplementary materials:  crystallographic information; 3D view; checkCIF report

## Figures and Tables

**Table 1:** Hydrogen-bond geometry (Å, °) *Cg*1, *Cg*7, *Cg*13 and *Cg*15 are the centroids of the C1*A*–C6*A*, C1*B*–C6*B*, C1*C*–C6*C* and C8*C*–C13*C* rings, respectively.

*D*—H⋯*A*	*D*—H	H⋯*A*	*D*⋯*A*	*D*—H⋯*A*
C10*A*—H10*A*⋯O1*C*^i^	0.93	2.52	3.4097 (16)	160
C10*C*—H10*C*⋯O1*B*^ii^	0.93	2.59	3.4374 (18)	151
C3*C*—H3*CA*⋯O1*A*^iii^	0.93	2.45	3.2287 (17)	141
C15*B*—H15*D*⋯O1*B*^ii^	0.96	2.43	3.3568 (18)	161
C16*A*—H16*A*⋯O1*A*^iv^	0.96	2.48	3.3934 (16)	159
C3*A*—H3*AA*⋯*Cg*13^v^	0.93	2.69	3.3996 (14)	134
C3*B*—H3*BA*⋯*Cg*13^vi^	0.93	2.77	3.3715 (14)	123
C15*A*—H15*B*⋯*Cg*15^v^	0.96	2.71	3.4915 (15)	139
C15*C*—H15*G*⋯*Cg*1^vii^	0.96	2.96	3.8656 (13)	158
C16*C*—H16*G*⋯*Cg*7^vi^	0.96	2.91	3.7432 (14)	145

Symmetry codes: (i) 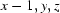; (ii) 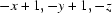; (iii) 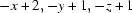; (iv) 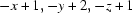; (v) 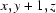; (vi) 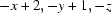; (vii) 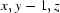.

**Fig. 1.**
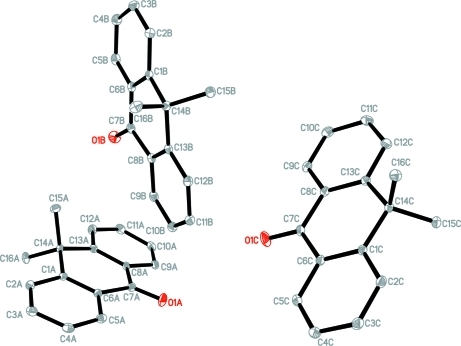
The asymmetric unit of the title compound, showing 30% probability displacement ellipsoids and the atom-numbering scheme. H atoms are omitted for clarity.

**Fig. 2.**
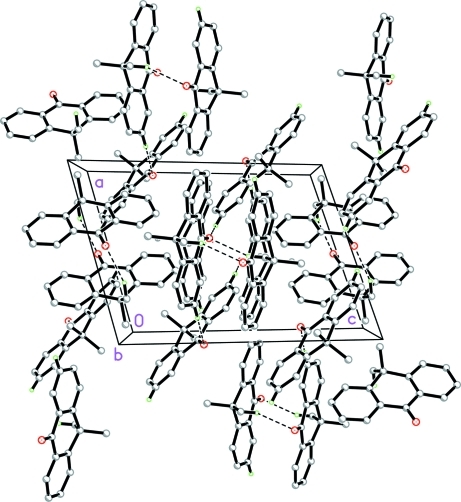
Crystal packing of the title compound, showing the hydrogen-bonding network (dashed lines). H atoms not involved in the hydrogen bond interactions are omitted for clarity.

## supplementary crystallographic information

### Comment

Anthrone is a tricyclic aromatic hydrocarbon which is used for a popular cellulose assay and in the colorometric determination of carbohydrates (Trevelyan, 1952).   The crystal structures of anthrone (Srivastava, 1964), 10-bromoanthrone (Destro *et al.*, 1973), 9,10-dimethylanthracene (Iball & Low, 1974), benzylideneanthrone at 193 K (Ghosh *et al.*, 1993), 10-(2-methylbenzylid- ene)anthrone (Zhou *et al.*,  2004), 10-(3,4-dimethoxybenzylidene) anthrone (Zhou *et al.*, 2005) and 10-(4-hydroxy-3-nitrobenzylidene) anthrone (Zhou *et al.*,  2007), have been reported.  In view of the importance of the title compound, (I), its crystal structure is reported here.

The asymmetric unit of the title compound, consists of three crystallographically independent 10,10-Dimethylanthrone  molecules, (A, B & C), as shown in Fig. 1.  The bond lengths and angles of molecules A , B and C agree with each other and are within normal ranges (Allen *et al.*, 1987). The anthracene units are essentially planar with maximum deviations of 0.165 (1)Å for atom C3A (molecule A), 0.153 (1)Å for atom C14B (molecule B) and 0.045 (1)Å for atom C14C (molecule C). The two fused benzene rings, C1–C6 and C8–C13, make dihedral angles with the C1/C6–C8/C13–C14 plane of  5.74 (5)° and 3.85 (5)° in molecule A; 4.40 (6)° and 2.79 (6)° in molecule B; and 1.69 (6)° and 1.63 (6)° in molecule C.

In the crystal structure (Fig. 2), the molecules are linked through intermolecular C10—H10A···O1C, C10C—H10C···O1B, C3C—H3CA···O1A C15B—H15D···O1B and C16A—H16A···O1A hydrogen bonds. The structure is further stabilized by C—H···π interactions (Table 1), involving C1A–C6A (centroid Cg1), C1B–C6B (centroid Cg7), C1C–C6C (centroid Cg13) and C8C–C13C (centroid Cg15).

### Experimental

The title compound was obtained as a gift sample from R. L. Fine Chem, Bangalore, India. X-ray quality crystals were obtained by slow evaporation from a methanol solution  (m. p.: 349–352 K).

### Refinement

All hydrogen atoms were positioned geometrically [C–H = 0.93 or 0.96Å] and were refined using a riding model, with *U*_iso_(H) = 1.2 or 1.5 *U*_eq_(C). A rotating group model was applied to the methyl groups.

### Crystal data

**Table:**

C_16_H_14_O	*Z* = 6
*M**_r_* = 222.27	*F*(000) = 708
Triclinic, *P*1	*D*_x_ = 1.278 Mg m^−^^3^
Hall symbol: -P 1	Mo *K*α radiation, λ = 0.71073 Å
*a* = 11.2438 (6) Å	Cell parameters from 9906 reflections
*b* = 12.1105 (6) Å	θ = 2.8–32.7°
*c* = 15.1025 (8) Å	µ = 0.08 mm^−^^1^
α = 107.955 (1)°	*T* = 100 K
β = 98.734 (1)°	Block, orange
γ = 111.764 (1)°	0.62 × 0.31 × 0.27 mm
*V* = 1732.47 (16) Å^3^	

### Data collection

**Table:**

Bruker APEX DUO CCD area-detector diffractometer	10016 independent reflections
Radiation source: fine-focus sealed tube	8420 reflections with *I* > 2σ(*I*)
graphite	*R*_int_ = 0.028
φ and ω scans	θ_max_ = 30.0°, θ_min_ = 1.5°
Absorption correction: multi-scan (*SADABS*; Bruker, 2009)	*h* = −15→15
*T*_min_ = 0.953, *T*_max_ = 0.979	*k* = −17→17
35076 measured reflections	*l* = −21→21

### Refinement

**Table:**

Refinement on *F*^2^	Primary atom site location: structure-invariant direct methods
Least-squares matrix: full	Secondary atom site location: difference Fourier map
*R*[*F*^2^ > 2σ(*F*^2^)] = 0.044	Hydrogen site location: inferred from neighbouring sites
*wR*(*F*^2^) = 0.142	H-atom parameters constrained
*S* = 1.07	*w* = 1/[σ^2^(*F*_o_^2^) + (0.0807*P*)^2^ + 0.476*P*] where *P* = (*F*_o_^2^ + 2*F*_c_^2^)/3
10016 reflections	(Δ/σ)_max_ = 0.001
466 parameters	Δρ_max_ = 0.54 e Å^−^^3^
0 restraints	Δρ_min_ = −0.36 e Å^−^^3^

### Special details

**Table:**

Experimental. The crystal was placed in the cold stream of an Oxford Cryosystems Cobra open-flow nitrogen cryostat (Cosier & Glazer, 1986) operating at 100.0 (1) K.
Geometry. All s.u.'s (except the s.u. in the dihedral angle between two l.s. planes) are estimated using the full covariance matrix. The cell s.u.'s are taken into account individually in the estimation of s.u.'s in distances, angles and torsion angles; correlations between s.u.'s in cell parameters are only used when they are defined by crystal symmetry. An approximate (isotropic) treatment of cell s.u.'s is used for estimating s.u.'s involving l.s. planes.
Refinement. Refinement of F^2^ against ALL reflections. The weighted R-factor wR and goodness of fit S are based on F^2^, conventional R-factors R are based on F, with F set to zero for negative F^2^. The threshold expression of F^2^ > 2σ(F^2^) is used only for calculating R-factors(gt) etc. and is not relevant to the choice of reflections for refinement. R-factors based on F^2^ are statistically about twice as large as those based on F, and R- factors based on ALL data will be even larger.

### Fractional atomic coordinates and isotropic or equivalent isotropic displacement parameters (Å^2^)

**Table:**

	*x*	*y*	*z*	*U*_iso_*/*U*_eq_	
O1A	0.56816 (9)	0.79013 (8)	0.44954 (7)	0.02699 (19)	
C1A	0.68614 (10)	1.07682 (10)	0.39587 (7)	0.01479 (19)	
C2A	0.80724 (11)	1.18655 (11)	0.41974 (8)	0.0192 (2)	
H2AA	0.8086	1.2474	0.3940	0.023*	
C3A	0.92550 (11)	1.20656 (11)	0.48111 (8)	0.0218 (2)	
H3AA	1.0040	1.2814	0.4972	0.026*	
C4A	0.92729 (11)	1.11539 (12)	0.51856 (8)	0.0218 (2)	
H4AA	1.0068	1.1278	0.5584	0.026*	
C5A	0.80939 (11)	1.00627 (11)	0.49582 (8)	0.0194 (2)	
H5AA	0.8097	0.9447	0.5203	0.023*	
C6A	0.68883 (10)	0.98739 (10)	0.43599 (7)	0.01541 (19)	
C7A	0.56463 (11)	0.87236 (10)	0.41915 (7)	0.01671 (19)	
C8A	0.43582 (10)	0.86035 (10)	0.36546 (7)	0.01535 (19)	
C9A	0.31622 (11)	0.75993 (10)	0.35921 (8)	0.0189 (2)	
H9AA	0.3201	0.7045	0.3894	0.023*	
C10A	0.19306 (11)	0.74253 (10)	0.30882 (8)	0.0210 (2)	
H10A	0.1141	0.6768	0.3058	0.025*	
C11A	0.18879 (11)	0.82504 (11)	0.26257 (9)	0.0223 (2)	
H11A	0.1065	0.8137	0.2278	0.027*	
C12A	0.30651 (11)	0.92392 (11)	0.26805 (8)	0.0209 (2)	
H12A	0.3018	0.9771	0.2358	0.025*	
C13A	0.43266 (10)	0.94566 (10)	0.32106 (7)	0.01543 (19)	
C14A	0.55932 (10)	1.05499 (10)	0.32446 (7)	0.01520 (19)	
C15A	0.57639 (12)	1.01805 (12)	0.22108 (8)	0.0218 (2)	
H15A	0.5831	0.9378	0.2022	0.033*	
H15B	0.6565	1.0850	0.2215	0.033*	
H15C	0.5001	1.0085	0.1754	0.033*	
C16A	0.54310 (12)	1.18059 (10)	0.35169 (9)	0.0216 (2)	
H16A	0.5360	1.2064	0.4167	0.032*	
H16B	0.4634	1.1662	0.3068	0.032*	
H16C	0.6198	1.2477	0.3486	0.032*	
O1B	0.52412 (9)	0.74204 (9)	0.03541 (7)	0.02682 (19)	
C1B	0.69616 (10)	0.58076 (10)	−0.09660 (7)	0.01525 (19)	
C2B	0.70059 (11)	0.53353 (11)	−0.19275 (8)	0.0196 (2)	
H2BA	0.7581	0.4961	−0.2062	0.023*	
C3B	0.62091 (12)	0.54135 (11)	−0.26839 (8)	0.0226 (2)	
H3BA	0.6242	0.5077	−0.3318	0.027*	
C4B	0.53651 (12)	0.59936 (11)	−0.24945 (8)	0.0238 (2)	
H4BA	0.4842	0.6057	−0.2998	0.029*	
C5B	0.53076 (11)	0.64754 (11)	−0.15537 (8)	0.0210 (2)	
H5BA	0.4746	0.6868	−0.1424	0.025*	
C6B	0.60928 (10)	0.63766 (10)	−0.07907 (8)	0.01640 (19)	
C7B	0.59492 (10)	0.68598 (10)	0.01946 (8)	0.01716 (19)	
C8B	0.66776 (10)	0.66388 (10)	0.09737 (8)	0.01560 (19)	
C9B	0.64508 (11)	0.69874 (10)	0.18895 (8)	0.0186 (2)	
H9BA	0.5841	0.7329	0.1983	0.022*	
C10B	0.71228 (12)	0.68289 (11)	0.26493 (8)	0.0203 (2)	
H10B	0.6968	0.7061	0.3252	0.024*	
C11B	0.80371 (11)	0.63168 (11)	0.25022 (8)	0.0195 (2)	
H11B	0.8496	0.6208	0.3010	0.023*	
C12B	0.82647 (11)	0.59700 (10)	0.16019 (8)	0.0178 (2)	
H12B	0.8880	0.5634	0.1517	0.021*	
C13B	0.75874 (10)	0.61140 (9)	0.08158 (7)	0.01495 (18)	
C14B	0.78902 (10)	0.57404 (10)	−0.01569 (7)	0.01553 (19)	
C15B	0.77900 (13)	0.43618 (11)	−0.04709 (8)	0.0236 (2)	
H15D	0.6880	0.3751	−0.0599	0.035*	
H15E	0.8370	0.4322	0.0042	0.035*	
H15F	0.8057	0.4155	−0.1051	0.035*	
C16B	0.93459 (11)	0.67118 (12)	−0.00016 (9)	0.0234 (2)	
H16D	0.9418	0.7573	0.0205	0.035*	
H16E	0.9563	0.6495	−0.0603	0.035*	
H16F	0.9958	0.6669	0.0490	0.035*	
O1C	0.95232 (9)	0.45834 (8)	0.29323 (7)	0.0297 (2)	
C1C	1.10277 (10)	0.24592 (9)	0.31380 (7)	0.01459 (18)	
C2C	1.20914 (11)	0.25001 (10)	0.37943 (8)	0.0185 (2)	
H2CA	1.2281	0.1791	0.3644	0.022*	
C3C	1.28635 (12)	0.35724 (11)	0.46597 (8)	0.0230 (2)	
H3CA	1.3558	0.3574	0.5083	0.028*	
C4C	1.26000 (13)	0.46478 (11)	0.48958 (8)	0.0247 (2)	
H4CA	1.3111	0.5365	0.5479	0.030*	
C5C	1.15738 (12)	0.46413 (10)	0.42563 (8)	0.0201 (2)	
H5CA	1.1402	0.5361	0.4408	0.024*	
C6C	1.07904 (10)	0.35551 (9)	0.33796 (7)	0.01501 (19)	
C7C	0.97047 (10)	0.36071 (10)	0.27270 (8)	0.01652 (19)	
C8C	0.88406 (10)	0.24447 (10)	0.18236 (7)	0.01471 (18)	
C9C	0.77830 (11)	0.24772 (11)	0.12168 (8)	0.0180 (2)	
H9CA	0.7660	0.3225	0.1388	0.022*	
C10C	0.69261 (11)	0.14160 (12)	0.03723 (8)	0.0219 (2)	
H10C	0.6232	0.1445	−0.0028	0.026*	
C11C	0.71192 (12)	0.03012 (11)	0.01295 (8)	0.0226 (2)	
H11C	0.6544	−0.0424	−0.0434	0.027*	
C12C	0.81653 (11)	0.02660 (11)	0.07228 (8)	0.0193 (2)	
H12C	0.8282	−0.0485	0.0545	0.023*	
C13C	0.90527 (10)	0.13340 (10)	0.15846 (7)	0.01460 (18)	
C14C	1.01912 (10)	0.12450 (10)	0.22050 (7)	0.01462 (18)	
C15C	0.95875 (11)	0.00734 (10)	0.24858 (8)	0.0194 (2)	
H15G	0.8980	0.0188	0.2848	0.029*	
H15H	1.0297	0.0013	0.2880	0.029*	
H15I	0.9111	−0.0707	0.1904	0.029*	
C16C	1.11272 (11)	0.10352 (11)	0.15848 (8)	0.0197 (2)	
H16G	1.1483	0.1751	0.1397	0.030*	
H16H	1.0627	0.0248	0.1011	0.030*	
H16I	1.1853	0.0977	0.1962	0.030*	

### Atomic displacement parameters (Å^2^)

**Table:**

	*U*^11^	*U*^22^	*U*^33^	*U*^12^	*U*^13^	*U*^23^
O1A	0.0275 (4)	0.0230 (4)	0.0374 (5)	0.0127 (4)	0.0079 (4)	0.0198 (4)
C1A	0.0167 (5)	0.0150 (4)	0.0130 (4)	0.0075 (4)	0.0056 (3)	0.0051 (3)
C2A	0.0200 (5)	0.0183 (5)	0.0180 (5)	0.0061 (4)	0.0066 (4)	0.0081 (4)
C3A	0.0170 (5)	0.0229 (5)	0.0190 (5)	0.0043 (4)	0.0056 (4)	0.0058 (4)
C4A	0.0184 (5)	0.0271 (6)	0.0172 (5)	0.0105 (4)	0.0033 (4)	0.0058 (4)
C5A	0.0209 (5)	0.0228 (5)	0.0177 (5)	0.0127 (4)	0.0052 (4)	0.0086 (4)
C6A	0.0175 (5)	0.0157 (4)	0.0144 (4)	0.0087 (4)	0.0055 (3)	0.0056 (4)
C7A	0.0194 (5)	0.0157 (4)	0.0175 (4)	0.0092 (4)	0.0065 (4)	0.0074 (4)
C8A	0.0180 (5)	0.0129 (4)	0.0158 (4)	0.0078 (4)	0.0061 (3)	0.0049 (3)
C9A	0.0222 (5)	0.0138 (4)	0.0201 (5)	0.0069 (4)	0.0087 (4)	0.0062 (4)
C10A	0.0184 (5)	0.0155 (5)	0.0231 (5)	0.0048 (4)	0.0073 (4)	0.0031 (4)
C11A	0.0163 (5)	0.0212 (5)	0.0250 (5)	0.0082 (4)	0.0035 (4)	0.0050 (4)
C12A	0.0197 (5)	0.0203 (5)	0.0243 (5)	0.0103 (4)	0.0049 (4)	0.0100 (4)
C13A	0.0167 (5)	0.0143 (4)	0.0163 (4)	0.0081 (4)	0.0057 (4)	0.0054 (4)
C14A	0.0174 (5)	0.0140 (4)	0.0154 (4)	0.0071 (4)	0.0051 (3)	0.0071 (3)
C15A	0.0227 (5)	0.0260 (5)	0.0160 (5)	0.0088 (4)	0.0062 (4)	0.0098 (4)
C16A	0.0231 (5)	0.0137 (5)	0.0302 (6)	0.0101 (4)	0.0077 (4)	0.0091 (4)
O1B	0.0291 (4)	0.0303 (5)	0.0296 (4)	0.0230 (4)	0.0088 (3)	0.0105 (4)
C1B	0.0144 (4)	0.0130 (4)	0.0182 (4)	0.0055 (4)	0.0038 (3)	0.0073 (4)
C2B	0.0201 (5)	0.0191 (5)	0.0195 (5)	0.0086 (4)	0.0060 (4)	0.0078 (4)
C3B	0.0231 (5)	0.0219 (5)	0.0181 (5)	0.0060 (4)	0.0034 (4)	0.0084 (4)
C4B	0.0216 (5)	0.0219 (5)	0.0217 (5)	0.0059 (4)	−0.0016 (4)	0.0096 (4)
C5B	0.0174 (5)	0.0182 (5)	0.0257 (5)	0.0078 (4)	0.0014 (4)	0.0091 (4)
C6B	0.0146 (4)	0.0130 (4)	0.0205 (5)	0.0058 (4)	0.0030 (4)	0.0066 (4)
C7B	0.0159 (5)	0.0145 (4)	0.0215 (5)	0.0078 (4)	0.0046 (4)	0.0069 (4)
C8B	0.0146 (4)	0.0124 (4)	0.0193 (5)	0.0058 (4)	0.0051 (4)	0.0058 (4)
C9B	0.0189 (5)	0.0156 (5)	0.0227 (5)	0.0089 (4)	0.0087 (4)	0.0067 (4)
C10B	0.0241 (5)	0.0176 (5)	0.0193 (5)	0.0088 (4)	0.0092 (4)	0.0068 (4)
C11B	0.0204 (5)	0.0189 (5)	0.0184 (5)	0.0079 (4)	0.0044 (4)	0.0080 (4)
C12B	0.0169 (5)	0.0177 (5)	0.0207 (5)	0.0090 (4)	0.0057 (4)	0.0084 (4)
C13B	0.0137 (4)	0.0124 (4)	0.0178 (4)	0.0050 (3)	0.0050 (3)	0.0056 (3)
C14B	0.0157 (4)	0.0165 (4)	0.0176 (4)	0.0095 (4)	0.0059 (3)	0.0076 (4)
C15B	0.0354 (6)	0.0232 (5)	0.0222 (5)	0.0209 (5)	0.0113 (5)	0.0106 (4)
C16B	0.0154 (5)	0.0323 (6)	0.0236 (5)	0.0090 (4)	0.0069 (4)	0.0139 (5)
O1C	0.0302 (5)	0.0166 (4)	0.0363 (5)	0.0140 (4)	−0.0027 (4)	0.0047 (3)
C1C	0.0147 (4)	0.0139 (4)	0.0151 (4)	0.0053 (4)	0.0041 (3)	0.0071 (3)
C2C	0.0186 (5)	0.0176 (5)	0.0204 (5)	0.0084 (4)	0.0032 (4)	0.0097 (4)
C3C	0.0217 (5)	0.0213 (5)	0.0212 (5)	0.0069 (4)	−0.0018 (4)	0.0096 (4)
C4C	0.0271 (6)	0.0174 (5)	0.0193 (5)	0.0053 (4)	−0.0030 (4)	0.0048 (4)
C5C	0.0231 (5)	0.0137 (4)	0.0196 (5)	0.0068 (4)	0.0024 (4)	0.0056 (4)
C6C	0.0155 (4)	0.0128 (4)	0.0159 (4)	0.0052 (4)	0.0034 (3)	0.0066 (4)
C7C	0.0162 (5)	0.0130 (4)	0.0193 (5)	0.0058 (4)	0.0033 (4)	0.0069 (4)
C8C	0.0141 (4)	0.0150 (4)	0.0156 (4)	0.0058 (4)	0.0043 (3)	0.0077 (4)
C9C	0.0172 (5)	0.0199 (5)	0.0205 (5)	0.0092 (4)	0.0051 (4)	0.0114 (4)
C10C	0.0185 (5)	0.0279 (6)	0.0196 (5)	0.0102 (4)	0.0023 (4)	0.0114 (4)
C11C	0.0210 (5)	0.0235 (5)	0.0156 (5)	0.0070 (4)	0.0005 (4)	0.0039 (4)
C12C	0.0205 (5)	0.0183 (5)	0.0170 (5)	0.0089 (4)	0.0043 (4)	0.0047 (4)
C13C	0.0146 (4)	0.0155 (4)	0.0143 (4)	0.0063 (4)	0.0049 (3)	0.0069 (4)
C14C	0.0162 (4)	0.0134 (4)	0.0151 (4)	0.0075 (4)	0.0040 (3)	0.0061 (3)
C15C	0.0229 (5)	0.0142 (4)	0.0201 (5)	0.0072 (4)	0.0046 (4)	0.0080 (4)
C16C	0.0195 (5)	0.0222 (5)	0.0205 (5)	0.0123 (4)	0.0072 (4)	0.0079 (4)

### Geometric parameters (Å, °)

**Table:**

O1A—C7A	1.2286 (13)	C9B—H9BA	0.9300
C1A—C2A	1.4013 (14)	C10B—C11B	1.3957 (16)
C1A—C6A	1.4016 (14)	C10B—H10B	0.9300
C1A—C14A	1.5309 (14)	C11B—C12B	1.3871 (15)
C2A—C3A	1.3908 (16)	C11B—H11B	0.9300
C2A—H2AA	0.9300	C12B—C13B	1.4040 (14)
C3A—C4A	1.3920 (17)	C12B—H12B	0.9300
C3A—H3AA	0.9300	C13B—C14B	1.5281 (14)
C4A—C5A	1.3800 (16)	C14B—C15B	1.5430 (15)
C4A—H4AA	0.9300	C14B—C16B	1.5503 (15)
C5A—C6A	1.4061 (14)	C15B—H15D	0.9600
C5A—H5AA	0.9300	C15B—H15E	0.9600
C6A—C7A	1.4787 (14)	C15B—H15F	0.9600
C7A—C8A	1.4800 (15)	C16B—H16D	0.9600
C8A—C13A	1.4020 (14)	C16B—H16E	0.9600
C8A—C9A	1.4051 (14)	C16B—H16F	0.9600
C9A—C10A	1.3804 (16)	O1C—C7C	1.2280 (13)
C9A—H9AA	0.9300	C1C—C6C	1.3998 (14)
C10A—C11A	1.3945 (17)	C1C—C2C	1.4075 (14)
C10A—H10A	0.9300	C1C—C14C	1.5254 (14)
C11A—C12A	1.3867 (16)	C2C—C3C	1.3861 (15)
C11A—H11A	0.9300	C2C—H2CA	0.9300
C12A—C13A	1.4037 (15)	C3C—C4C	1.3939 (17)
C12A—H12A	0.9300	C3C—H3CA	0.9300
C13A—C14A	1.5235 (14)	C4C—C5C	1.3836 (16)
C14A—C16A	1.5359 (14)	C4C—H4CA	0.9300
C14A—C15A	1.5487 (14)	C5C—C6C	1.4043 (14)
C15A—H15A	0.9600	C5C—H5CA	0.9300
C15A—H15B	0.9600	C6C—C7C	1.4818 (14)
C15A—H15C	0.9600	C7C—C8C	1.4786 (14)
C16A—H16A	0.9600	C8C—C13C	1.4004 (14)
C16A—H16B	0.9600	C8C—C9C	1.4076 (14)
C16A—H16C	0.9600	C9C—C10C	1.3800 (15)
O1B—C7B	1.2292 (13)	C9C—H9CA	0.9300
C1B—C6B	1.4015 (14)	C10C—C11C	1.3931 (17)
C1B—C2B	1.4032 (14)	C10C—H10C	0.9300
C1B—C14B	1.5254 (14)	C11C—C12C	1.3889 (15)
C2B—C3B	1.3912 (15)	C11C—H11C	0.9300
C2B—H2BA	0.9300	C12C—C13C	1.4047 (14)
C3B—C4B	1.3889 (17)	C12C—H12C	0.9300
C3B—H3BA	0.9300	C13C—C14C	1.5260 (14)
C4B—C5B	1.3814 (17)	C14C—C15C	1.5485 (14)
C4B—H4BA	0.9300	C14C—C16C	1.5494 (14)
C5B—C6B	1.4058 (14)	C15C—H15G	0.9600
C5B—H5BA	0.9300	C15C—H15H	0.9600
C6B—C7B	1.4795 (15)	C15C—H15I	0.9600
C7B—C8B	1.4801 (14)	C16C—H16G	0.9600
C8B—C13B	1.4035 (14)	C16C—H16H	0.9600
C8B—C9B	1.4082 (14)	C16C—H16I	0.9600
C9B—C10B	1.3801 (16)		
			
C2A—C1A—C6A	117.35 (10)	C11B—C10B—H10B	120.4
C2A—C1A—C14A	120.03 (9)	C12B—C11B—C10B	120.30 (10)
C6A—C1A—C14A	122.55 (9)	C12B—C11B—H11B	119.9
C3A—C2A—C1A	121.50 (10)	C10B—C11B—H11B	119.9
C3A—C2A—H2AA	119.3	C11B—C12B—C13B	121.56 (10)
C1A—C2A—H2AA	119.3	C11B—C12B—H12B	119.2
C2A—C3A—C4A	120.49 (10)	C13B—C12B—H12B	119.2
C2A—C3A—H3AA	119.8	C8B—C13B—C12B	117.68 (9)
C4A—C3A—H3AA	119.8	C8B—C13B—C14B	122.66 (9)
C5A—C4A—C3A	119.08 (10)	C12B—C13B—C14B	119.63 (9)
C5A—C4A—H4AA	120.5	C1B—C14B—C13B	113.08 (8)
C3A—C4A—H4AA	120.5	C1B—C14B—C15B	109.74 (9)
C4A—C5A—C6A	120.61 (10)	C13B—C14B—C15B	109.38 (8)
C4A—C5A—H5AA	119.7	C1B—C14B—C16B	107.61 (8)
C6A—C5A—H5AA	119.7	C13B—C14B—C16B	107.82 (8)
C1A—C6A—C5A	120.93 (10)	C15B—C14B—C16B	109.10 (9)
C1A—C6A—C7A	121.15 (9)	C14B—C15B—H15D	109.5
C5A—C6A—C7A	117.90 (9)	C14B—C15B—H15E	109.5
O1A—C7A—C6A	121.08 (10)	H15D—C15B—H15E	109.5
O1A—C7A—C8A	121.12 (10)	C14B—C15B—H15F	109.5
C6A—C7A—C8A	117.80 (9)	H15D—C15B—H15F	109.5
C13A—C8A—C9A	120.68 (10)	H15E—C15B—H15F	109.5
C13A—C8A—C7A	121.17 (9)	C14B—C16B—H16D	109.5
C9A—C8A—C7A	118.15 (9)	C14B—C16B—H16E	109.5
C10A—C9A—C8A	120.83 (10)	H16D—C16B—H16E	109.5
C10A—C9A—H9AA	119.6	C14B—C16B—H16F	109.5
C8A—C9A—H9AA	119.6	H16D—C16B—H16F	109.5
C9A—C10A—C11A	119.01 (10)	H16E—C16B—H16F	109.5
C9A—C10A—H10A	120.5	C6C—C1C—C2C	117.59 (9)
C11A—C10A—H10A	120.5	C6C—C1C—C14C	122.92 (9)
C12A—C11A—C10A	120.43 (10)	C2C—C1C—C14C	119.48 (9)
C12A—C11A—H11A	119.8	C3C—C2C—C1C	121.57 (10)
C10A—C11A—H11A	119.8	C3C—C2C—H2CA	119.2
C11A—C12A—C13A	121.57 (10)	C1C—C2C—H2CA	119.2
C11A—C12A—H12A	119.2	C2C—C3C—C4C	120.06 (10)
C13A—C12A—H12A	119.2	C2C—C3C—H3CA	120.0
C8A—C13A—C12A	117.44 (10)	C4C—C3C—H3CA	120.0
C8A—C13A—C14A	122.81 (9)	C5C—C4C—C3C	119.55 (10)
C12A—C13A—C14A	119.69 (9)	C5C—C4C—H4CA	120.2
C13A—C14A—C1A	112.91 (8)	C3C—C4C—H4CA	120.2
C13A—C14A—C16A	109.53 (8)	C4C—C5C—C6C	120.42 (10)
C1A—C14A—C16A	109.86 (9)	C4C—C5C—H5CA	119.8
C13A—C14A—C15A	107.85 (8)	C6C—C5C—H5CA	119.8
C1A—C14A—C15A	107.88 (8)	C1C—C6C—C5C	120.78 (10)
C16A—C14A—C15A	108.70 (9)	C1C—C6C—C7C	121.40 (9)
C14A—C15A—H15A	109.5	C5C—C6C—C7C	117.82 (9)
C14A—C15A—H15B	109.5	O1C—C7C—C8C	120.98 (10)
H15A—C15A—H15B	109.5	O1C—C7C—C6C	121.04 (10)
C14A—C15A—H15C	109.5	C8C—C7C—C6C	117.98 (9)
H15A—C15A—H15C	109.5	C13C—C8C—C9C	120.82 (9)
H15B—C15A—H15C	109.5	C13C—C8C—C7C	121.05 (9)
C14A—C16A—H16A	109.5	C9C—C8C—C7C	118.12 (9)
C14A—C16A—H16B	109.5	C10C—C9C—C8C	120.96 (10)
H16A—C16A—H16B	109.5	C10C—C9C—H9CA	119.5
C14A—C16A—H16C	109.5	C8C—C9C—H9CA	119.5
H16A—C16A—H16C	109.5	C9C—C10C—C11C	118.83 (10)
H16B—C16A—H16C	109.5	C9C—C10C—H10C	120.6
C6B—C1B—C2B	117.54 (9)	C11C—C10C—H10C	120.6
C6B—C1B—C14B	122.94 (9)	C12C—C11C—C10C	120.45 (10)
C2B—C1B—C14B	119.46 (9)	C12C—C11C—H11C	119.8
C3B—C2B—C1B	121.51 (10)	C10C—C11C—H11C	119.8
C3B—C2B—H2BA	119.2	C11C—C12C—C13C	121.79 (10)
C1B—C2B—H2BA	119.2	C11C—C12C—H12C	119.1
C4B—C3B—C2B	120.13 (10)	C13C—C12C—H12C	119.1
C4B—C3B—H3BA	119.9	C8C—C13C—C12C	117.14 (9)
C2B—C3B—H3BA	119.9	C8C—C13C—C14C	123.28 (9)
C5B—C4B—C3B	119.62 (10)	C12C—C13C—C14C	119.57 (9)
C5B—C4B—H4BA	120.2	C1C—C14C—C13C	113.28 (8)
C3B—C4B—H4BA	120.2	C1C—C14C—C15C	108.61 (8)
C4B—C5B—C6B	120.39 (10)	C13C—C14C—C15C	108.91 (8)
C4B—C5B—H5BA	119.8	C1C—C14C—C16C	108.38 (8)
C6B—C5B—H5BA	119.8	C13C—C14C—C16C	108.09 (8)
C1B—C6B—C5B	120.78 (10)	C15C—C14C—C16C	109.54 (8)
C1B—C6B—C7B	121.12 (9)	C14C—C15C—H15G	109.5
C5B—C6B—C7B	118.08 (9)	C14C—C15C—H15H	109.5
O1B—C7B—C6B	120.70 (10)	H15G—C15C—H15H	109.5
O1B—C7B—C8B	121.46 (10)	C14C—C15C—H15I	109.5
C6B—C7B—C8B	117.83 (9)	H15G—C15C—H15I	109.5
C13B—C8B—C9B	120.44 (10)	H15H—C15C—H15I	109.5
C13B—C8B—C7B	121.31 (9)	C14C—C16C—H16G	109.5
C9B—C8B—C7B	118.25 (9)	C14C—C16C—H16H	109.5
C10B—C9B—C8B	120.80 (10)	H16G—C16C—H16H	109.5
C10B—C9B—H9BA	119.6	C14C—C16C—H16I	109.5
C8B—C9B—H9BA	119.6	H16G—C16C—H16I	109.5
C9B—C10B—C11B	119.22 (10)	H16H—C16C—H16I	109.5
C9B—C10B—H10B	120.4		
			
C6A—C1A—C2A—C3A	−0.26 (15)	C8B—C9B—C10B—C11B	0.04 (16)
C14A—C1A—C2A—C3A	−177.26 (9)	C9B—C10B—C11B—C12B	−0.10 (16)
C1A—C2A—C3A—C4A	1.80 (16)	C10B—C11B—C12B—C13B	−0.32 (16)
C2A—C3A—C4A—C5A	−1.52 (16)	C9B—C8B—C13B—C12B	−0.83 (15)
C3A—C4A—C5A—C6A	−0.26 (16)	C7B—C8B—C13B—C12B	178.22 (9)
C2A—C1A—C6A—C5A	−1.52 (14)	C9B—C8B—C13B—C14B	−178.93 (9)
C14A—C1A—C6A—C5A	175.40 (9)	C7B—C8B—C13B—C14B	0.12 (15)
C2A—C1A—C6A—C7A	176.71 (9)	C11B—C12B—C13B—C8B	0.77 (15)
C14A—C1A—C6A—C7A	−6.38 (14)	C11B—C12B—C13B—C14B	178.93 (10)
C4A—C5A—C6A—C1A	1.81 (15)	C6B—C1B—C14B—C13B	10.95 (14)
C4A—C5A—C6A—C7A	−176.48 (10)	C2B—C1B—C14B—C13B	−171.94 (9)
C1A—C6A—C7A—O1A	175.70 (10)	C6B—C1B—C14B—C15B	133.38 (10)
C5A—C6A—C7A—O1A	−6.02 (15)	C2B—C1B—C14B—C15B	−49.51 (12)
C1A—C6A—C7A—C8A	−4.97 (14)	C6B—C1B—C14B—C16B	−108.02 (11)
C5A—C6A—C7A—C8A	173.31 (9)	C2B—C1B—C14B—C16B	69.10 (12)
O1A—C7A—C8A—C13A	−172.58 (10)	C8B—C13B—C14B—C1B	−8.51 (14)
C6A—C7A—C8A—C13A	8.10 (14)	C12B—C13B—C14B—C1B	173.42 (9)
O1A—C7A—C8A—C9A	6.77 (15)	C8B—C13B—C14B—C15B	−131.14 (10)
C6A—C7A—C8A—C9A	−172.56 (9)	C12B—C13B—C14B—C15B	50.80 (12)
C13A—C8A—C9A—C10A	0.37 (15)	C8B—C13B—C14B—C16B	110.33 (11)
C7A—C8A—C9A—C10A	−178.98 (9)	C12B—C13B—C14B—C16B	−67.73 (12)
C8A—C9A—C10A—C11A	1.02 (16)	C6C—C1C—C2C—C3C	1.30 (16)
C9A—C10A—C11A—C12A	−0.68 (16)	C14C—C1C—C2C—C3C	−178.80 (10)
C10A—C11A—C12A—C13A	−1.05 (17)	C1C—C2C—C3C—C4C	−0.35 (17)
C9A—C8A—C13A—C12A	−2.02 (15)	C2C—C3C—C4C—C5C	−0.68 (18)
C7A—C8A—C13A—C12A	177.31 (9)	C3C—C4C—C5C—C6C	0.72 (18)
C9A—C8A—C13A—C14A	−179.17 (9)	C2C—C1C—C6C—C5C	−1.25 (15)
C7A—C8A—C13A—C14A	0.16 (15)	C14C—C1C—C6C—C5C	178.85 (9)
C11A—C12A—C13A—C8A	2.37 (16)	C2C—C1C—C6C—C7C	179.17 (9)
C11A—C12A—C13A—C14A	179.62 (10)	C14C—C1C—C6C—C7C	−0.73 (15)
C8A—C13A—C14A—C1A	−10.51 (13)	C4C—C5C—C6C—C1C	0.27 (16)
C12A—C13A—C14A—C1A	172.40 (9)	C4C—C5C—C6C—C7C	179.86 (10)
C8A—C13A—C14A—C16A	−133.28 (10)	C1C—C6C—C7C—O1C	−178.21 (10)
C12A—C13A—C14A—C16A	49.63 (12)	C5C—C6C—C7C—O1C	2.20 (16)
C8A—C13A—C14A—C15A	108.59 (11)	C1C—C6C—C7C—C8C	2.14 (14)
C12A—C13A—C14A—C15A	−68.50 (12)	C5C—C6C—C7C—C8C	−177.45 (9)
C2A—C1A—C14A—C13A	−169.52 (9)	O1C—C7C—C8C—C13C	179.49 (10)
C6A—C1A—C14A—C13A	13.65 (13)	C6C—C7C—C8C—C13C	−0.86 (14)
C2A—C1A—C14A—C16A	−46.94 (12)	O1C—C7C—C8C—C9C	−1.52 (15)
C6A—C1A—C14A—C16A	136.23 (10)	C6C—C7C—C8C—C9C	178.13 (9)
C2A—C1A—C14A—C15A	71.40 (12)	C13C—C8C—C9C—C10C	0.14 (16)
C6A—C1A—C14A—C15A	−105.43 (11)	C7C—C8C—C9C—C10C	−178.85 (10)
C6B—C1B—C2B—C3B	−0.66 (16)	C8C—C9C—C10C—C11C	0.35 (16)
C14B—C1B—C2B—C3B	−177.93 (10)	C9C—C10C—C11C—C12C	−0.69 (17)
C1B—C2B—C3B—C4B	1.40 (17)	C10C—C11C—C12C—C13C	0.55 (17)
C2B—C3B—C4B—C5B	−0.92 (17)	C9C—C8C—C13C—C12C	−0.28 (14)
C3B—C4B—C5B—C6B	−0.26 (17)	C7C—C8C—C13C—C12C	178.68 (9)
C2B—C1B—C6B—C5B	−0.53 (15)	C9C—C8C—C13C—C14C	179.19 (9)
C14B—C1B—C6B—C5B	176.64 (9)	C7C—C8C—C13C—C14C	−1.85 (15)
C2B—C1B—C6B—C7B	177.92 (9)	C11C—C12C—C13C—C8C	−0.06 (15)
C14B—C1B—C6B—C7B	−4.92 (15)	C11C—C12C—C13C—C14C	−179.55 (10)
C4B—C5B—C6B—C1B	1.00 (16)	C6C—C1C—C14C—C13C	−1.78 (13)
C4B—C5B—C6B—C7B	−177.50 (10)	C2C—C1C—C14C—C13C	178.33 (9)
C1B—C6B—C7B—O1B	176.03 (10)	C6C—C1C—C14C—C15C	−122.91 (10)
C5B—C6B—C7B—O1B	−5.48 (15)	C2C—C1C—C14C—C15C	57.19 (12)
C1B—C6B—C7B—C8B	−4.30 (15)	C6C—C1C—C14C—C16C	118.17 (10)
C5B—C6B—C7B—C8B	174.19 (9)	C2C—C1C—C14C—C16C	−61.73 (12)
O1B—C7B—C8B—C13B	−173.65 (10)	C8C—C13C—C14C—C1C	3.08 (13)
C6B—C7B—C8B—C13B	6.68 (14)	C12C—C13C—C14C—C1C	−177.46 (9)
O1B—C7B—C8B—C9B	5.42 (15)	C8C—C13C—C14C—C15C	124.05 (10)
C6B—C7B—C8B—C9B	−174.25 (9)	C12C—C13C—C14C—C15C	−56.49 (12)
C13B—C8B—C9B—C10B	0.44 (16)	C8C—C13C—C14C—C16C	−117.03 (10)
C7B—C8B—C9B—C10B	−178.64 (10)	C12C—C13C—C14C—C16C	62.43 (12)

### Hydrogen-bond geometry (Å, °)

**Table:**

Cg1, Cg7, Cg13 and Cg15 are the centroids of the C1A–C6A, C1B–C6B, C1C–C6C and C8C–C13C rings, respectively.

**Table:**

*D*—H···*A*	*D*—H	H···*A*	*D*···*A*	*D*—H···*A*
C10A—H10A···O1C^i^	0.93	2.52	3.4097 (16)	160
C10C—H10C···O1B^ii^	0.93	2.59	3.4374 (18)	151
C3C—H3CA···O1A^iii^	0.93	2.45	3.2287 (17)	141
C15B—H15D···O1B^ii^	0.96	2.43	3.3568 (18)	161
C16A—H16A···O1A^iv^	0.96	2.48	3.3934 (16)	159
C3A—H3AA···Cg13^v^	0.93	2.69	3.3996 (14)	134
C3B—H3BA···Cg13^vi^	0.93	2.77	3.3715 (14)	123
C15A—H15B···Cg15^v^	0.96	2.71	3.4915 (15)	139
C15C—H15G···Cg1^vii^	0.96	2.96	3.8656 (13)	158
C16C—H16G···Cg7^vi^	0.96	2.91	3.7432 (14)	145

Symmetry codes: (i) *x*−1, *y*, *z*; (ii) −*x*+1, −*y*+1, −*z*; (iii) −*x*+2, −*y*+1, −*z*+1; (iv) −*x*+1, −*y*+2, −*z*+1; (v) *x*, *y*+1, *z*; (vi) −*x*+2, −*y*+1, −*z*; (vii) *x*, *y*−1, *z*.
